# Structural Significance of Lipid Diversity as Studied by Small Angle Neutron and X-ray Scattering

**DOI:** 10.3390/membranes5030454

**Published:** 2015-09-21

**Authors:** Norbert Kučerka, Frederick A. Heberle, Jianjun Pan, John Katsaras

**Affiliations:** 1 Frank Laboratory of Neutron Physics, Joint Institute for Nuclear Research, 141980 Dubna—Moscow Region, Russia; 2 Department of Physical Chemistry of Drugs, Faculty of Pharmacy, Comenius University, 832 32 Bratislava, Slovakia; 3 Biology & Soft Matter Division, Oak Ridge National Laboratory, Oak Ridge, TN 37831, USA; E-Mail: heberlefa@ornl.gov; 4 Joint Institute for Neutron Sciences, Oak Ridge National Laboratory, Oak Ridge, TN 37831-6453, USA; 5 Department of Physics, University of South Florida, Tampa, FL 33620, USA; E-Mail: panj@usf.edu; 6 Department of Physics and Astronomy, University of Tennessee, Knoxville, TN 37996-1200, USA

**Keywords:** lipidome, bilayer, structure, X-ray scattering, neutron scattering, lipid area

## Abstract

We review recent developments in the rapidly growing field of membrane biophysics, with a focus on the structural properties of single lipid bilayers determined by different scattering techniques, namely neutron and X-ray scattering. The need for accurate lipid structural properties is emphasized by the sometimes conflicting results found in the literature, even in the case of the most studied lipid bilayers. Increasingly, accurate and detailed structural models require more experimental data, such as those from contrast varied neutron scattering and X-ray scattering experiments that are jointly refined with molecular dynamics simulations. This experimental and computational approach produces robust bilayer structural parameters that enable insights, for example, into the interplay between collective membrane properties and its components (e.g., hydrocarbon chain length and unsaturation, and lipid headgroup composition). From model studies such as these, one is better able to appreciate how a real biological membrane can be tuned by balancing the contributions from the lipid’s different moieties (e.g., acyl chains, headgroups, backbones, *etc.*).

## 1. Introduction

Biological membranes play a crucial role in defining the properties of cells and biological tissues. Membranes form a natural barrier that separates the cytosol from its extracellular environment. However, these complex mesoscopic assemblies also possess functions, which are far more elaborate than those of a simple passive permeability barrier populated with proteins. Instead, biomembranes are functional dynamic machines that are central to a host of biological processes, including the transport of materials, and cell integrity, recognition, adhesion and signaling, to name but a few. In general, the different organelle membranes serve different functions and consequently have different structural properties. Indeed, as structure is often associated with function, the myriad processes taking place in these membranes are reflected in the lipidome’s size and diversity [1]. For example, the thermodynamic phase of multicomponent lipid mixtures plays a key role in determining the membrane’s physicochemical properties [2].

Biological membranes consist mainly of lipids and proteins, where it is widely accepted that the membrane’s underlying structure is imparted by the lipid bilayer. The double-layered membrane structure was first proposed at the beginning of 20th century [3] by demonstrating that lipids extracted from red blood cells covered an area that was approximately twice the cell surface area from which they were extracted. Subsequent characterization of this structure as a permeability barrier [4], together with observations of thermotropic phase transitions [5], eventually led to the notion of the fluid mosaic model [6], which subsequently became the dominant paradigm for biological membrane structure. Over the years, tremendous efforts have been expended in studying lipid bilayer structure and dynamics in hopes of understanding the functional mechanisms taking place at membrane interfaces. The notion of nanometer-size lipid rafts influencing the membrane’s biophysical environment is the most current model for the crowded plasma membrane [7]. The structure-function relationship is also of great interest for pharmaceutical applications hoping to facilitate cell-to-cell communication, direct proteins to their destinations, regulate the uptake and transport of cholesterol or genetic material by individual cells, as well as the ability to control docking sites for opportunistic viruses to bind with, and infect individual cells.

Due to the compositional complexity of biological membranes, the physical properties and functional roles of individual lipid species are difficult to determine. In order to gain insight into the roles of the different lipid species, it is necessary to study model membrane systems. For example, in eukaryotic cells the predominant lipid species are glycerol-based phospholipids, namely phosphatidylcholine (PC), phosphatidylethanolamine (PE), phosphatidylserine (PS), phosphatidylglycerol (PG), phosphatidylinositol (PI), and cardiolipin (CL), while major phospholipids observed in prokaryotic membranes are PE, PG, and CL [8]. Under neutral pH conditions, the PC and PE headgroups are electrically neutral, while the PS, PG, and PI headgroups have a net negative charge. A given mixture of neutral and anionic lipids in a membrane thus confers a surface charge density that not only influences the membrane’s permeability to ions and charged molecules, but can also affect membrane protein function. For example, bacteria are known to adjust their PE/PG ratio when exposed to toxic organic solvents, a scenario that presumably alters their surface charge density, thus minimizing permeability to solutes (e.g., protons and other ions) while preserving bilayer integrity [9]. The membrane’s thermodynamic phase is primarily determined by the chemical composition of its lipids (e.g., hydrocarbon chains and polar headgroups), and its capacity to attract water. For example, in the case of PE bilayers which interact with fewer water molecules, the main gel-to-liquid disordered phase transition temperature increases by as much as 30 °C, compared to their counterpart PC bilayers [10]. A mechanism for modifying membrane surface area by changing the PC/PE ratio appears to be associated with liver disease [11] and heart myocytes [12] in mammalian cells. In addition to headgroup diversity, each lipid species exhibits a characteristic, yet fairly diverse, fatty acid composition. For example, the inability to maintain proper levels of membrane unsaturation can result in biological malfunction, as has been recently recognized in gestational diabetes mellitus [13]. Therefore, in order to better understand the cell membrane, there is a clear need for the careful and precise characterization of its individual lipid components.

Another important parameter is molecular geometry, where for example the relatively small PE headgroup imposes a membrane curvature that may be necessary for accommodating certain proteins, and which may in turn modulate their function [14]. We should note, however, that in the current manuscript we will only discuss lipids that form bilayers and not other geometries such hexagonal, cubic, *etc.* To first order, this means that the average molecular shape should resemble a right circular cylinder. Moreover, we focus on reviewing the structural properties of single component lipid bilayers in their fully hydrated fluid phase, which are believed to be the most biologically relevant.

Molecular dynamics (MD) simulations, especially atomistic simulations, can provide quantitative details that surpass experimental data. An enormous development in the field, that is driven, for the most part, by increased computing power and parallel algorithms, has enabled larger and more complex systems to be studied that better approximate biology [15,16,17]. Structural results extracted from MD simulations can readily interrogate lipid moieties with specific biological relevance. It is also recognized that the accuracy of MD simulations is directly associated with the force fields used. It is therefore necessary to first verify such simulations with reliable experimental results [18].

Area per lipid has been frequently used as a key parameter when assessing the validity of MD simulations. In fact, the importance of lipid area is such that simulators routinely constrained lateral area, or surface tension, in their simulations [19]. The obvious criticism for such an approach, however, stems from the fact that area per lipid—same as electron or neutron scattering density profiles—is not a direct experimental result, but is model dependent [20]. In an ideal world, the ability of a given force field to simulate a fluid bilayer should be scrutinized without the need for a model, by directly comparing simulation and experiment, as has been proposed recently [21], and used with good success [18,22,23,24,25,26,27,28,29,30,31,32].

It has become evident over the recent past that there is much synergy between simulation and experiment, where simulation results aid the design of models for the analysis of experimental data, and in turn, experimental results help to improve the force fields used in simulations. Regardless of how the comparison is made (*i.e.*, in reciprocal or real space), it is always beneficial to include as much experimental data as possible. An example of this was the discrepancy between fluid DOPC area per lipid values that were determined using stand-alone X-ray or neutron scattering, and those determined using a joint data refinement approach [33]. Here we focus on bilayer parameters obtained using the recently developed technique that maximizes the use of complementary data information.

There are a number of experimental techniques suitable for studying biomembranes at the microscopic level. For example, microscopic techniques can provide information on nanomechanics [34], phases and local structures [35], from the micron to the nanometer length scale. Generally, samples studied under biologically relevant conditions give rise to lower resolution data (e.g., various optical microscopies), with some exceptions (e.g., AFM [36]). In contrast, scattering techniques allow for the *in situ* manipulation of samples, while providing quantitative data on the global distribution of different structural features (e.g., size, shape, and correlation lengths) [37]. Among the often-used spectroscopic (e.g., electron paramagnetic resonance, NMR, fluorescence) and calorimetric techniques, X-ray and neutron scattering have over the years proven to be two of the most widely used techniques in structural biology, biophysics and materials science [38]. Numerous water-soluble proteins have been crystallized and their structures resolved at atomic resolutions. On the other hand, due to the intrinsic disorder in biomimetic samples—a disorder most likely important for the proper function of biology systems—membrane soluble proteins have proven, thus far, difficult to crystallize. For such systems, broad statistical distributions, rather than sharp delta functions, are used to best describe them [39]. This is especially true for the structure of biomimetic lipid membranes in their biologically relevant liquid crystalline state.

Neutron and X-ray scattering are similar in that both techniques are capable of providing dynamical and structural information [40]. However, the principal differences between the two techniques are in their interactions with matter. X-rays are electromagnetic waves that primarily interact with electrons, and their amplitude of X-ray scattering length increases in a simple way with atomic number *Z* (*i.e.*, *a* = Z *r_e_*, where electron radius *r_e_ =* 2.8179 × 10^−5^ Å), while neutrons are particles that interact with atomic nuclei, and neutron scattering amplitudes depend in a complex manner on the mass, spin and energy levels of the nuclei that they scatter from. Additionally, neutron scattering from individual moieties of a macromolecular complex can be enhanced or reduced through the unique ability of neutrons to distinguish between hydrogen and its stable isotope. For example, by specific deuterium-labeling, it is possible to measure bilayer conformational changes and organization in both the perpendicular and lateral directions in a membrane, including the detection and characterization of nanoscopic lipid domains [41,42,43,44,45]. The recent advances in X-ray and neutron scattering methods are increasingly providing us with unique access to the much touted structure-function relationship in biomembranes that is universally sought out in biology and pharmacology.

## 2. Lipid Membrane Structure Determination

Advances in colloid and interface science have stimulated a renewed interest in the study of lipid–water systems. At the same time, much progress has been made in the analysis of small-angle X-ray and neutron scattering data. The popularity of small-angle scattering for the study of biologically relevant materials stems from the fact that it provides detailed information on the size, shape and conformation of molecular assemblies in solution. As a result, structural biophysics has taken advantage of recent developments to accurately determine the structure of lipid bilayers. An example of this is the joint refinement [46] of X-ray and neutron scattering data, which has been rethought in terms of improving the values of lipid areas [33]. (A comprehensive overview of the various model membrane platforms used to study biomembranes has been recently compiled by Heberle *et al.* [47].)

**Figure 1:**
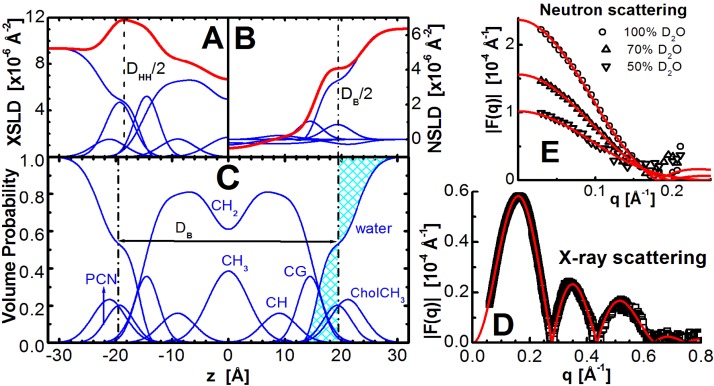
Illustration of lipid bilayer structure determination through the joint refinement of X-ray and neutron scattering data. The scattering density profile (SDP) representation of a bilayer in real space is shown on the left, where the top panels show X-ray scattering length density (XSLD) with amplitudes calculated from the number of electrons and electron radius (**A**), and neutron scattering length density (NSLD) based on neutron coherent scattering amplitudes (**B**) of lipid component distributions (see e.g., [33] for detailed description). The total scattering length densities are denoted by the thick red lines. Panel (**C**) shows volume probability distributions, where the total probability is equal to 1 at each point across the bilayer, and the location where the shaded areas are equal defines the Gibbs dividing surface between the lipid bilayer and the water phase (effectively *D_B_*). Graphs on the right show the experimentally determined X-ray (**D**) and contrast varied neutron (**E**) scattering form factors (points), together with the best fits to the data (solid lines).

The models of X-ray scattering length density (XSLD) and neutron scattering length density (NSLD) emphasize different, but complementary features of the bilayer (e.g., compare Figure 1A,B). It follows that a combined approach describes the structural features accentuated by each technique, but in a manner that the data are analyzed simultaneously. This is illustrated in the way the lipid molecular area *A* is determined, a parameter central to bilayer structure. For both X-ray and neutron scattering, *A* is calculated using the bilayer’s thickness and additional volumetric information. However, it should be emphasized that the two scattering techniques are sensitive to different bilayer thicknesses. The thickness best resolved by X-rays is the distance between the electron density maxima found in the lipid headgroup region, *D_HH_*, while in the case of neutron scattering, the high contrast between the protiated lipid and deuterated water accurately defines the total bilayer thickness, *D_B_*. Even though they are the two most robust experimentally determined parameters, *D_HH_* and *D_B_* are not directly comparable, and neither measure on its own contains all of the desired bilayer structural information. Instead, models are used to determine the remaining structural parameters. The simultaneous analysis of X-ray and neutron scattering data results in robust structural parameters that describe key bilayer features.

The main objective of models is to obtain structural information. Area per lipid is one of the most important parameters needed to accurately describe bilayer structure, and lipid-lipid and lipid-protein interactions in biomembranes. However, *A* is not easily determined, and is only obtained if additional information is provided to constrain the many parameters used in any realistic model of a bilayer. It should be emphasized, however, that this is not a criticism for the use of models. On the contrary, the unique advantage of models is that information from other experiments can be used to refine them [48]. For example, total lipid volume, *V_L_*, one of the most accurately experimentally obtained parameters [49,50,51], can be used to reduce the total number of variables. Even though the experimentally obtained scattering contains information about the bilayer’s structure in the z direction (*i.e.*, along the bilayer normal), the inclusion of *V_L_* allows for the evaluation of structure in the lateral direction, namely *A*.

The experimentally obtained X-ray (Figure 1D) and neutron (Figure 1E) scattering data are generally fitted with model calculated curves in an iterative procedure, until the difference between the two is minimized. The final set of parameters is then assigned to model the bilayer structure. A variety of structural models have been applied to membranes, and most of them were specific to X-ray or neutron scattering data [48,52,53]. However, more robust methods of analyses have also been implemented that address X-ray and neutron scattering data, simultaneously [33,54]. Their advantage is that a single model is used to analyze both datasets. Note, that although different parsing schemes have been used to describe lipids of different chemical compositions, each must fulfill the main principle of space filling models, namely that all component probabilities add up to unity at each point across the bilayer (Figure 1C). The model is then a set of parameters that are related to each other in a consistent manner (e.g., *V_total_ = D_total_* × *A = D_hydrocarbon_* × *A+D_polar_* × *A*). This means that the SDP model includes both X-ray and neutron bilayer thicknesses (Figure 1A,B). We will focus on data analysis using this approach.

## 3. Lipid Chain Length

Biological activities of surface-active compounds are known to depend on lipid acyl chain length [55]. An exciting possibility is that the biological membrane has at its disposal a wide range of lipid lengths to stimulate membrane proteins at different locations. Experimental evidence for this has been reported in the case of sarcoplasmic reticulum Ca^2+^-transporting ATPase reconstituted into lipid bilayers [56]. Enzymatic activity was found to be maximal in bilayers composed of medium length (18-carbon) lipids, while it decreased significantly in both short- (14-carbon) and long-chain (22-carbon) lipid bilayers. It is now generally accepted that the function, insertion, orientation and subcellular localization of integral membrane proteins are affected by the membrane’s hydrophobic thickness and bending rigidity, which are dictated by the membrane’s lipid, cholesterol and protein composition [57,58,59]. The non-random distribution of these molecules within a membrane leads to the wide range of membrane thicknesses observed in the different organelles of eukaryotic cells [60]. As a result of the structural flexibility of lipid hydrocarbon chains, a membrane can adjust its thickness to minimize unfavorable thermodynamic interactions between water and a protein’s hydrophobic region, a process known as hydrophobic matching [61].

**Figure 2:**
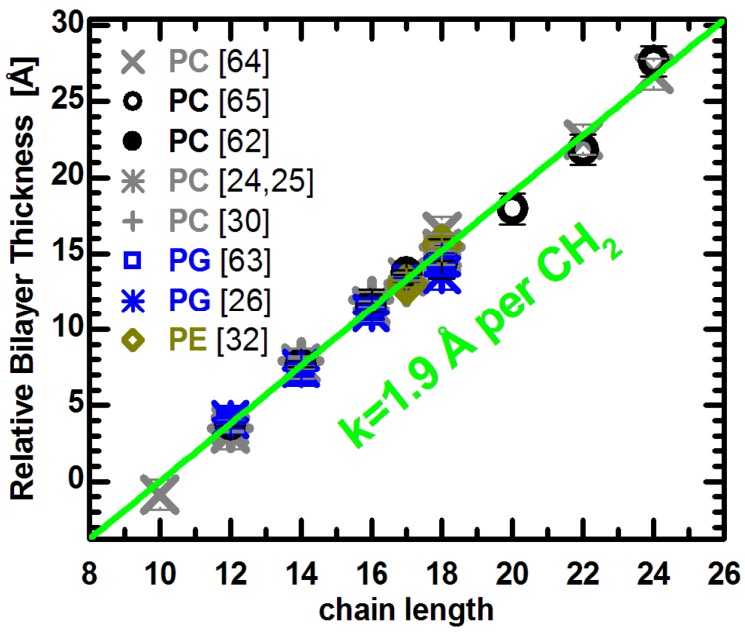
Relative bilayer thicknesses as a function of chain length for lipids with different headgroups, acyl chain unsaturation and temperature. Changes to the bilayer thickness were taken from the literature (see text), and each series was shifted vertically to lie on the green line. The slope of the linear function was adopted from previous results [62,63], and its origin was set arbitrarily to *n* = 10.

Intuitively, the length of a bilayer’s acyl chains affects, to first order, bilayer thickness. Although the reported absolute thickness values for given lipid bilayers vary widely [39], their relative changes should be independent of the experimental method (e.g., X-ray *vs.* neutron scattering) and model (e.g., *D_HH_ vs. D_B_*) used. We have therefore collected a series of bilayer thickness parameters reported in the literature, and shifted each series vertically so that they all lie on a common straight line. The effect of acyl chain length can then be assessed for lipids of various chemical compositions, as shown in Figure 2.

Lewis and Engelman reported that bilayer thickness increased linearly in PC bilayers with saturated acyl chains ranging from 12 to 18 carbons in length, and for monounsaturated chains with 18 to 24 carbons [64]. It is important to note that all measurements were performed at the same reduced temperature (*i.e.*, relative to the lipid melting transition temperature), and therefore required correction before being compared at the same temperature. We used thermal thickness expansion coefficients published for saturated lipids [62], and the average *k_DB_* = −0.059 Å/deg for unsaturated lipids. The applied correction does not change the reported linearity of thickness changes, though it does slightly increase the slope. Importantly, both lipid groups are well fit by a line with a slope of 1.9 Å per CH_2_ group (note the distance between methylenes corresponds to half this number since the bilayer is spanned by two lipid molecules) [62,63]. The same linear relationship can be used to fit the thickness of unsaturated PC bilayers reported in [65], although a small quadratic deviation was originally proposed. Finally, the relative changes to the bilayer thickness of saturated, monounsaturated, and mixed chain PC lipids obtained experimentally [62] and by MD simulations [24,25,30], all lie on the same line. In addition, the latter data points were averaged over several temperatures, as they showed no thermal effect to the chain length imposed thickness changes (see e.g., Figure 8B in [62]).

It is interesting to compare the PC data to lipids with different headgroup compositions. The thickness changes reported for PG lipids with different acyl chain length and unsaturation has been determined from recent molecular structures obtained experimentally [63] and by simulations [26]. The PG results also agree with the linear relationship shown for PC bilayers. Together with results from PE bilayers [32], they suggest a common linear dependence of changes to bilayer thickness as a function of acyl chain length. This is independent of whether the bilayers are composed of lipids with different headgroups, or different acyl chain unsaturation. In other words, the addition of CH_2_ group results in a *universal* thickness change for fully hydrated fluid bilayers, regardless of their chemical make-up.

Figure 2 supports the notion that a change in the acyl chain length increases bilayer thickness similarly in the different bilayers. However, this is only part of the story. Another significant change takes place in the plane of the bilayer, namely to the area per lipid [66]. As a first approximation, one can estimate the behavior of *A* from the fact that bilayer thickness increases linearly with each additional carbon, and so does lipid volume. On the other hand, though lipid volume seems to change linearly as a function of acyl chain length, it is important to note its temperature dependence. From the slope of the fit at T = 30 °C, the volume of a CH_2_ group is estimated at 27.8 Å^3^, and expands to *V_CH2_* = 28.9 Å^3^ at T = 65 °C [50,51]. A simple division of the slopes corresponding to changes in volume and thickness results in a constant area per lipid of 58.5 Å^2^ and 60.8 Å^2^ at T = 30 °C and 65 °C, respectively. However, these values turn out to be too small when compared to experimental values [62], demonstrating that it is not possible to simplify changes in the lateral direction in the same way that one does in the transverse direction. It therefore seems that lipid areas are strongly dependent on the acyl chain and headgroup compositions.

**Figure 3:**
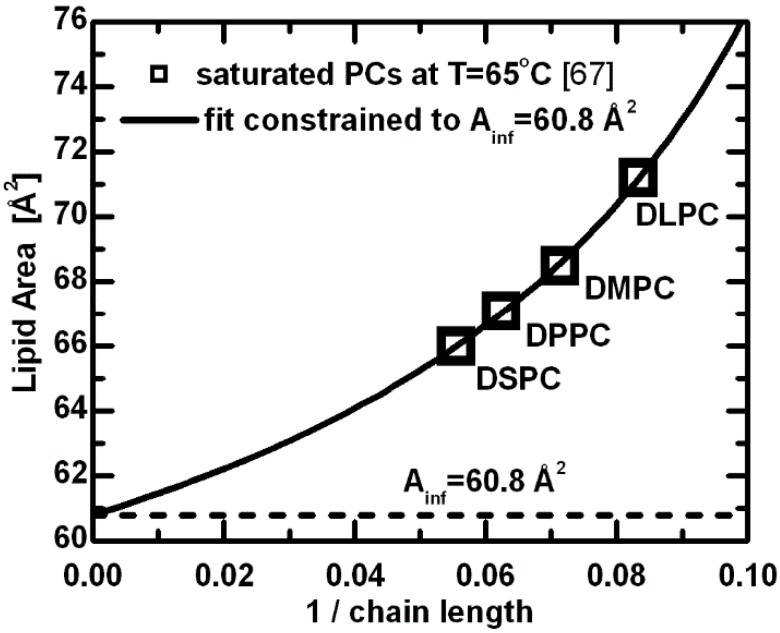
Lipid areas for a series of saturated PC lipids at T = 65 °C [67]. The hyperbolic function derived from the free energy description was used to fit the original data with a constraint of *A_inf_* (area per lipid with the infinitely long chains) equal to the limiting area per lipid estimated from the linear dependence of volumetric [50,51] and bilayer thickness data [62,63].

The origin of this discrepancy between bilayer thickness and area per lipid, and how they relate to acyl chain length stems from the delicate balance of forces that are responsible for minimizing the system’s total energy, and includes both headgroup and hydrocarbon chain interactions. A simple formulation of the free energy for a planar bilayer involves attractive components that are the result of hydrophobic forces within the hydrocarbon chain region, headgroup dipolar interactions, and most likely, other interactions [67]. These variables are in addition to the effects resulting from temperature and acyl chain length. The repulsive components are equally complex, including steric interactions, hydration forces and entropic effects due to acyl chain confinement [67]. When scrutinizing the consequences of increased acyl chain length, the attractive van der Waals forces between hydrocarbon tails probably play the most important role in determining the area per lipid—they contribute directly to a decreased lipid area as a function of increasing chain length. This is clearly shown in the hyperbolic nature of lipid area dependence (Figure 3), which decreases with increasing acyl chain length, eventually reaching a limiting value [67]. The latter represents an effective area for a given lipid headgroup. The limiting case corresponds to the mode of interactions with fully extended acyl chains, where further additions of methylene groups contribute only to the bilayer thickness. The above estimated constant lipid area can, to a first approximation, be understood as the limiting value for PC lipids with infinitely long acyl chains.

It is important to note that the previous discussion did not account for thermal effects which give rise to another essential interaction within the bilayer, namely trans-gauche isomerizations. The probability of trans-gauche isomerization in acyl chains increases with increasing temperature and has the opposite effect of the van der Waals force. In addition, the arbitrary shifting of bilayer thickness for the different lipids masks the differences in their absolute values—differences that are rooted in the compositional variability of the acyl chains and headgroups. To better appreciate these effects, we will return to the absolute values in the other sections of this review.

## 4. Lipid Chain Unsaturation

**Figure 4:**
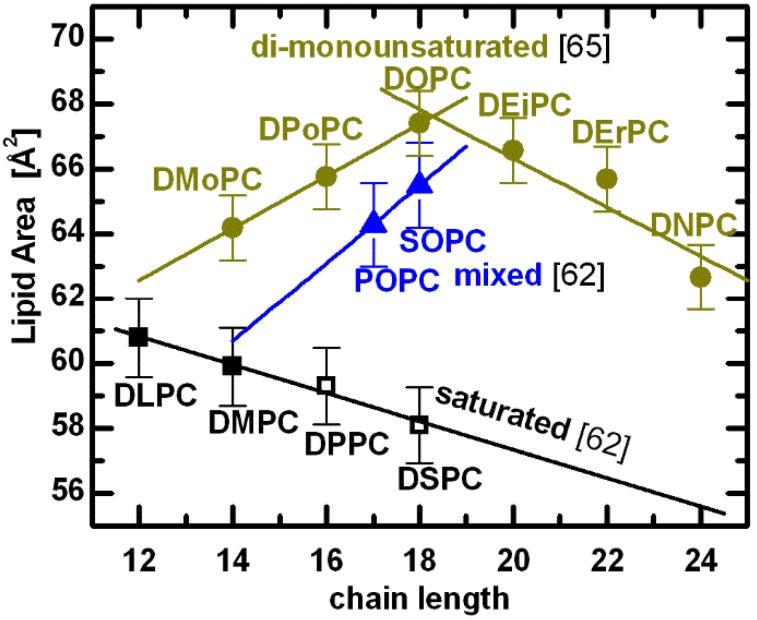
A compilation of PC lipid areas at T = 30 °C [62,65]. Shown are experimentally determined molecular areas for lipids composed of two saturated acyl chains (black: DiLauroylPC, DiMyristoylPC, DiPalmitoylPC, and DiStearoylPC, respectively), a saturated *sn-1* chain and a mono-*cis*-unsaturated *sn-2* chain (blue: PalmitoylOleoylPC, and StearoylOleoylPC, respectively), and two mono-*cis*-unsaturated chains (yellow: DiMyristoleoylPC, DiPalmitoleoylPC, DiOleoylPC, DiEicosenoylPC, DiErucoylPC, and DiNervonoylPC, respectively). Points denoted by open symbols were extrapolated from fluid phase data using expansivity parameters, as described in the text.

Changes to a lipid’s hydrocarbon chain length have been shown to affect the intrabilayer interactions, which are responsible for controlling bilayer thickness and lipid area. Interestingly, while the relative bilayer thickness appears to be affected equally for different lipids, in the case of area per lipid experimental results suggest differences between the chain length dependencies for lipids containing saturated *vs.* unsaturated fatty acid chains (note, we discuss *cis* unsaturation). A decrease in lipid area as a function of increasing chain length, shown in Figure 3, implies that for saturated chain lipids the largest contribution arises from van der Waals interactions. Over a limited range of chain lengths this behavior can be simplified to a linear function, as shown in Figure 4. Note that the comparison is done at T = 30 °C—for lipids with a higher melting temperature (*i.e.*, T*_M_* > 30 °C, DPPC and DSPC), fluid phase data were extrapolated to 30 °C using published thermal area expansivities [62].

A remarkable result is observed for lipids with double bonds in their acyl chains. Acyl chain unsaturation perturbs hydrocarbon chain packing, resulting in increased chain disorder and a concomitant increase in lipid area. Even a single double bond in mixed acyl chain lipids increases significantly their area per lipid (e.g., POPC and SOPC in Figure 4). Moreover, the addition of two methylene groups to mixed chain lipids (*i.e.*, blue line connecting POPC and SOPC) results in an area *increase*, in contrast to the decrease experienced by lipids with saturated chains (black line). This suggests that rotational isomerization plays a much more pronounced role in lipid areas of mixed chain lipid bilayers, counteracting the effect of the attractive van der Waals interactions [62]. In fact, the perturbation induced by the double bond on neighboring saturated chains is evident when comparing the areas of lipids having 18-carbon chains—the difference between DOPC and SOPC is small, compared to the difference between SOPC and DSPC. In other words, the effect of introducing a single double bond (*i.e.*, SOPC) to one chain of a disaturated lipid (*i.e.*, DSPC) is several times greater than adding a double bond to the second chain (*i.e.*, DOPC) [68].

Interestingly, structural results for di-monounsaturated lipids reveal a nonlinear relationship between the area per lipid and acyl chain length [65]. As chain length increases, the area per lipid first increases, and then decreases, exhibiting an area maximum when *n* = 18 (yellow lines in Figure 4). Although surprising at first, the nonlinear behavior of lipid area can be explained in terms of double bond position. For *n* = 14 to 18 bilayers, the double bond is at the 9-*cis* position, while for *n* = 20, 22 and 24 bilayers, it is at the 11-*cis*, 13-*cis* and 15-*cis* positions, respectively. As previously discussed, increasing the hydrocarbon chain length results in increased van der Waals attraction, which in turn leads to ordering of the hydrocarbon chains, effectively reducing the area per lipid. However, lipid chain disorder also depends on double bond position [69], presumably having most effect when the double bond is located in the middle of the hydrocarbon chain [70].

**Figure 5:**
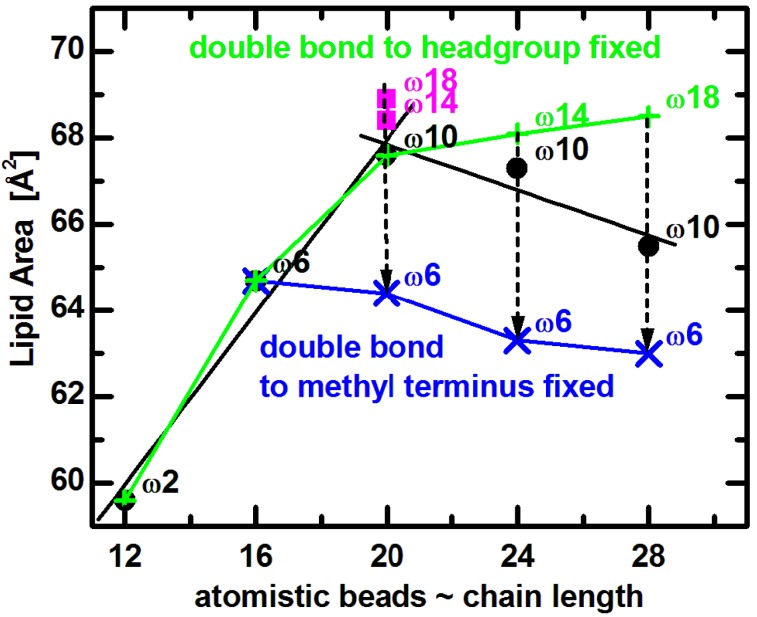
Coarse grained simulated areas for di-monounsaturated PC lipids at T = 30 °C [65]. Black solid dots correspond approximately to diC_12:1_PC, diC_16:1_PC, diC_20:1_PC, diC_24:1_PC, and diC_28:1_PC. The double bond position was fixed with respect to the lipid headgroup (green plus signs) or the methyl terminus (blue crosses), as described in the text. Dashed arrows indicate shifting of double bonds along the acyl chains in the direction away from lipid headgroup.

Comparing experimental results to MD simulations is most useful for understanding the effect of double bond position on lipid area. Coarse grained bilayer simulation results (Figure 5) reproduce the non-monotonic trend of lipid area dependence, and qualitatively confirm the same effect for lipids similar to those used in experiments [65]. (The small horizontal shift is due to the somewhat arbitrary mapping between the number of carbons and the coarse grained beads [71].) Focusing on trends, it is clear that *A* increases with increasing chain length when the double bond’s distance from the lipid headgroup remains constant (e.g., *n* = 12 to 20). On the other hand, for longer chain lipids (*n* = 20 to 28) with a fixed distance of the double bond with respect to the bilayer center, *A* *decreases* as a function of increasing hydrocarbon chain length. Most importantly, when the double bond is shifted away from the headgroup (dashed arrows in Figure 5), the lipid assumes a smaller area due to the lower disorder within its hydrocarbon chain region. It is obvious that changes in area follow a non-monotonic behavior, which levels off as the double bond gets closer to the headgroup [72,73]. Furthermore, keeping the double bond position at a fixed distance from the headgroup (9-cis) results in a lipid area increase over the entire range (green line in Figure 5), while *A* decreases when the double bond position is fixed with respect to the methyl terminus (at the ω6 position shown by blue line in Figure 5).

It is noteworthy that an effect similar to the addition of a *cis*-double bond on bilayer structural properties (*i.e.*, increased chain disorder and area per lipid) can be achieved by simple methyl substitution. In the case of di-phytanoylPC that contains four methyl groups along each of its hydrocarbon chains, an area increase of about 20 Å^2^ was observed [74,75,76,77]. The direct response of lipid area to changes in acyl chain length and composition emphasizes the importance of the hydrocarbon region’s structural properties, contributing to a better understanding of membrane-protein interactions. Importantly, this also shows that *A* is a good gauge for lateral interactions taking place within the bilayer.

## 5. Lipid Headgroup

**Figure 6:**
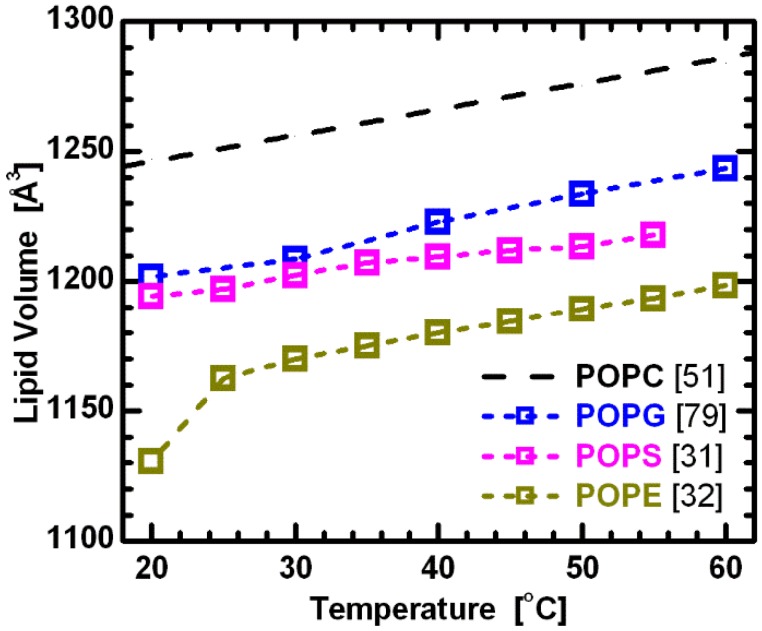
Molecular volumes for lipids composed of palmitoyl-oleoyl chains and different headgroups. All data corresponds to fluid phase bilayers, with the exception of POPE at 20 °C. The constant offsets between the linear temperature dependence of the different lipids suggest the validity of partially temperature-dependent segmental volumes [51], with headgroup volumes *V_PC_* = 331 Å^3^ [78], *V_PG_* = 291 Å^3^ [79], *V_PS_* = 278 Å^3^ [31], and *V_PE_* = 245Å^3^ [32].

Lipid bilayer structure is affected by acyl chain length and composition in both the transverse and lateral directions, effects that manifest themselves in lipid volume. Interestingly, however, Koenig and Gawrisch [51] showed that segmental volumes (*i.e.*, the volumes of CH, CH_2_ and CH_3_ groups) remain constant across PC lipids with different hydrocarbon chain lengths and degrees of unsaturation. They also speculated that this is true for phospholipids with different headgroups. Using this assumption, the comparison of volumetric results for lipids with the same hydrocarbon chains, but different headgroups (e.g., PC, PG, PS and PE), provides initial information regarding the headgroup’s influence on lipid bilayer structure. The more-or-less parallel curves in Figure 6 support this assumption, and at the same time suggest a constant headgroup volume for the different temperatures reported. It is again important to note that while PC and PE headgroups are electrically neutral under neutral pH conditions, PS and PG headgroups have a net negative charge. The system therefore includes Na^+^ counterions to neutralize the lipids, and experimental data were collected in 100 mM NaCl solution. This, of course, adds another level of complexity when comparing effects of different headgroups. Nevertheless, it is still interesting to compare the behavior of bilayers with different headgroup lipids.

**Figure 7:**
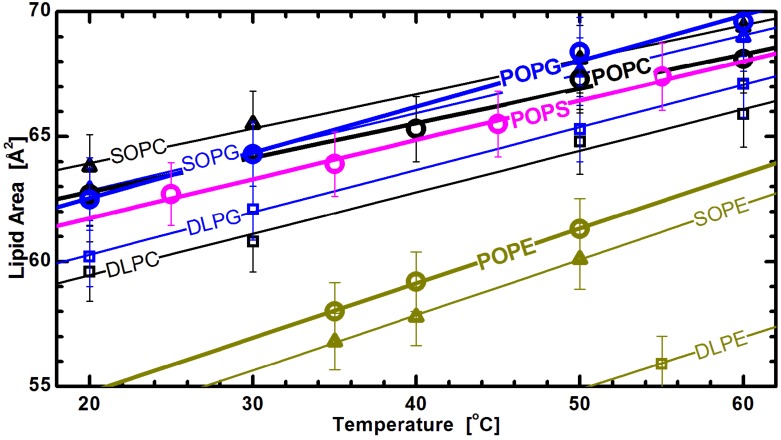
Lipid areas of different headgroup and acyl chain compositions as a function of temperature [32]. The straight lines are linear fits through the experimentally obtained data (note the lower temperature DLPE data are not shown for clarity), and suggest a similar thermal response for all different headgroups. Though they all exhibit the same thermal trend, PE areas are offset due to tighter headgroup packing, as discussed in the text.

While all the different lipids appear to exhibit a similar response to changes in acyl chain composition and temperature (see Figure 1 and Figure 6), the differences in bilayers with chemically different headgroups manifest themselves through the offsets of their structural parameters. For example, the larger thickness of PE bilayers can be attributed to the PE headgroup’s much smaller cross-sectional area [32]. Figure 7 shows lipid areas obtained for bilayers composed of different chain and headgroup combination lipids, with emphasis placed on those with an acyl chain motif found in animal cell membranes (*i.e.*, 1-palmitoyl-2-oleoyl, or PO). For these lipids, the modestly long acyl chains and single double bond make them a perfect candidate for studies of the effects discussed. The graph clearly shows the already mentioned common response to temperature changes for both saturated and mixed chain lipids. Interestingly, it also shows marginal differences between the lipid areas of PG and PS lipids, when compared to larger headgroup PC lipids (Figure 6). This observation highlights the importance of the glycerol-carbonyl backbone, common to these three headgroups, in dictating the nature of the interactions taking place in the headgroup region [79]. In contrast, however, the same does not hold true for PE headgroup lipids, despite sharing the same backbone. The uniqueness of PE lipids can be explained by the strong hydrogen bonding between its ${\text{NH}}_{3}^{+}$ and either the ${\text{PO}}_{2}^{-}$ or *C* = *O* group of its neighboring lipids [80]. This results in a tighter packing of PE headgroups, and consequently, radically reduced lipid areas. However, despite their smaller area per lipid, the thermal response of PE lipids follows the same trend as observed for other headgroups.

The location of lipid headgroups at the interface between the membrane’s hydrophobic interior and its aqueous exterior is central to their biological importance. Lipid headgroup diversity modulates the membrane’s surface electrostatic potential, which is in itself a crucial property for a wide range of biological processes [81,82]. For this reason, lipid areas likely play a central role in regulating membrane permeability and stability. For example, that the largest areas are observed for PG lipids—even though PG does not possess the largest headgroup—is consistent with the observation that the introduction of anionic PG lipids results in reduced membrane rupture pressure, which in turn affects membrane stability [83]. The larger areas of PG lipids can also play important roles in regulating protein translocation [84], modulating bacterial membrane permeability [85], and enhancing membrane protein folding [86]. In contrast, structural studies of PE bilayers show a low number of water molecules hydrating their headgroups (between 4 and 7, compared to ~12 for a typical fluid PC bilayer [87]). In fact, the steric exclusion interactions and strong hydrogen bonding between PE headgroups that are responsible for such low levels of hydration, are unique among the glycerophospholipids. When compared to PC headgroups with strong repulsive interactions below areas of ~48 Å^2^, and which prevent minimal packing of their acyl chains [88], the chains of DLPE, with its gel phase area ~41 Å^2^ [89], appear to achieve such packing. (We note that the minimal area of an all-trans chain is ~20 Å^2^ [90].) The fluid phase PE area most likely represents the packing limit for fluid chains that is dictated completely by chain interactions, as opposed to the prevailing head-head interactions found in the other classes of lipids [67].

In conclusion, the discussed structural results support the notion that hydrocarbon chains dominate the bilayer’s response to changes in temperature, while lipid headgroups govern bilayer packing. One can speculate that the differences in lipid headgroups provide biological membranes with a tool to *coarsely* tune the various inter-molecular interactions within the bilayer, while their properties are *fine*-tuned through the composition of their lipid acyl chains. The biological function of membranes is most likely then imparted by the structural diversity of the different lipid moieties found in the lipidome.
